# Development and pre-testing of the Patient Engagement In Research Scale (PEIRS) to assess the quality of engagement from a patient perspective

**DOI:** 10.1371/journal.pone.0206588

**Published:** 2018-11-01

**Authors:** Clayon B. Hamilton, Alison M. Hoens, Shanon McQuitty, Annette M. McKinnon, Kelly English, Catherine L. Backman, Tara Azimi, Negar Khodarahmi, Linda C. Li

**Affiliations:** 1 Department of Physical Therapy, University of British Columbia, Vancouver, BC, Canada; 2 Arthritis Research Canada, Richmond, BC, Canada; 3 Arthritis Patient Advisory Board, Arthritis Research Canada, Richmond, BC, Canada; 4 BC SUPPORT Unit, Vancouver, BC, Canada; 5 Department of Occupational Science and Occupational Therapy, University of British Columbia, Vancouver, BC, Canada; University of Montreal, CANADA

## Abstract

**Objectives:**

To develop and examine the content and face validity of the Patient Engagement In Research Scale (PEIRS) for assessing the quality of patient engagement in research projects from a patient partner perspective.

**Methods:**

Our team of researchers and patient partners conducted a mixed qualitative and quantitative study in three phases. Participants were English-speaking adult patients (including informal caregivers, family members, and friends) with varying experiences as partners in research projects in Canada. 1) Questionnaire items were generated following thematic analysis of in-depth interviews and published literature. 2) A three-round e-Delphi survey process via email correspondence was undertaken to refine and select the items for a provisional PEIRS. 3) Two rounds of cognitive interviewing elicited participants’ understanding and opinions of each item and the structure of the PEIRS.

**Results:**

One hundred and twenty items were generated from 18 interviews and organized across eight themes of meaningful engagement of patients in health research to form an initial questionnaire. The e-Delphi survey and cognitive interviewing each included 12 participants with a range of self-reported diseases, health-related conditions, and use of healthcare services. The e-Delphi survey yielded a 43-item provisional PEIRS. The PEIRS was then reduced to 37 items organized across seven themes after 1) refinement of problems in its instructions and items, and 2) the combining of two themes into one.

**Conclusions:**

We developed a 37-item self-reported questionnaire that has demonstrated preliminary content and face validity for assessing the quality of patient engagement in research.

## Introduction

Funding agencies, academic journals, and patient advocacy groups are promoting the engagement of patients in health research.[1–5] Patient engagement in research can be defined as patients taking part in hands-on, decision-making, and advisory activities beyond the role of study participants (for example, as co-researchers) at any and all stages of the research process.[6–8] The current study uses the term ‘patient engagement,’ which is commonly called ‘public and patient involvement’ in the United Kingdom [8] and may be known by other names in other contexts.

Patients are key partners in health research because their lived experiences and knowledge can enrich the quality, relevance, and impact of research when it takes into account their needs, values, and preferences.[4,9] The ultimate goal of patient engagement is to generate research that contributes to better healthcare service delivery, clinical outcomes, and population health.[3] Recent publications have reported several of its beneficial impacts on and outcomes for patient partners,[10] researchers,[11] and research projects.[12,13] For example, engaging patients has led to initial research priorities, study designs, study findings, as well as healthcare interventions, and knowledge translation strategies that align better with patient perspectives.[12,14]

Despite reported successes in engaging patients, systematic reviews have highlighted challenges such as tokenism and limited scope in the role and function of patients on research teams.[15,16] Tokenism has to do with failure to have a well-thought-out plan or strategy for truly engaging patients when they are part of a research team.[17] The evaluation of patients’ perspectives on their engagement is an important vehicle that can be used to move the quality of engagement on a continuum from tokenistic to meaningful.[17] At the meaningful end, the engagement of patients is planned, supported, valued, and conducted within a positive research environment, and is a rewarding experience for the patients.[6]

Studies over the last two decades have provided recommendations and principles for engaging patients in research. Some are specific to certain populations, research project types, or patients’ affiliations.[3,14,18,19] Other studies have developed frameworks or models to plan, undertake, evaluate, and report on patient engagement.[6,7,20–30] Effective methods of engaging patients could inform best practices for consistent and sustainable high standards in this area. However, there is limited evidence regarding the effectiveness of current methods, such as training workshops for patient partners and researchers, as interventions to enhance the engagement of patients in the research process.[31] A major barrier to gathering such evidence is that there are currently no validated measures to test the effectiveness of patient engagement interventions.[15]

Esmail et al. (2015) proposed dimensions for evaluating the context, process, and impact of patients’ and other stakeholders’ engagement in research.[32] Their literature review identified only qualitative assessments that covered some dimensions of impact.[32] Measures currently available may be unsuitable for evaluating patient engagement in research because of their target populations or articulated constructs.[19,33,34] The Quantitative Community Engagement Measure, for example, is based on 11 engagement principles from the community-engaged research literature.[19] Designed for quantifying the level of community engagement on a research project,[19] it primarily assesses community-academic partnerships. To our knowledge, it has not undergone any validation process to evaluate the quality of patients’ engagement throughout the research process.[19]

To advance the science and practice of patient engagement, it is critical to have valid and reliable measures to test the effectiveness of patient engagement interventions, such as training workshops, from the patients’ perspective. Hence, this study sought to develop and examine the content and face validity of a novel outcome measure for assessing the quality of patient engagement in research projects from a patient partner perspective.

## Methods

**Fig 1:**
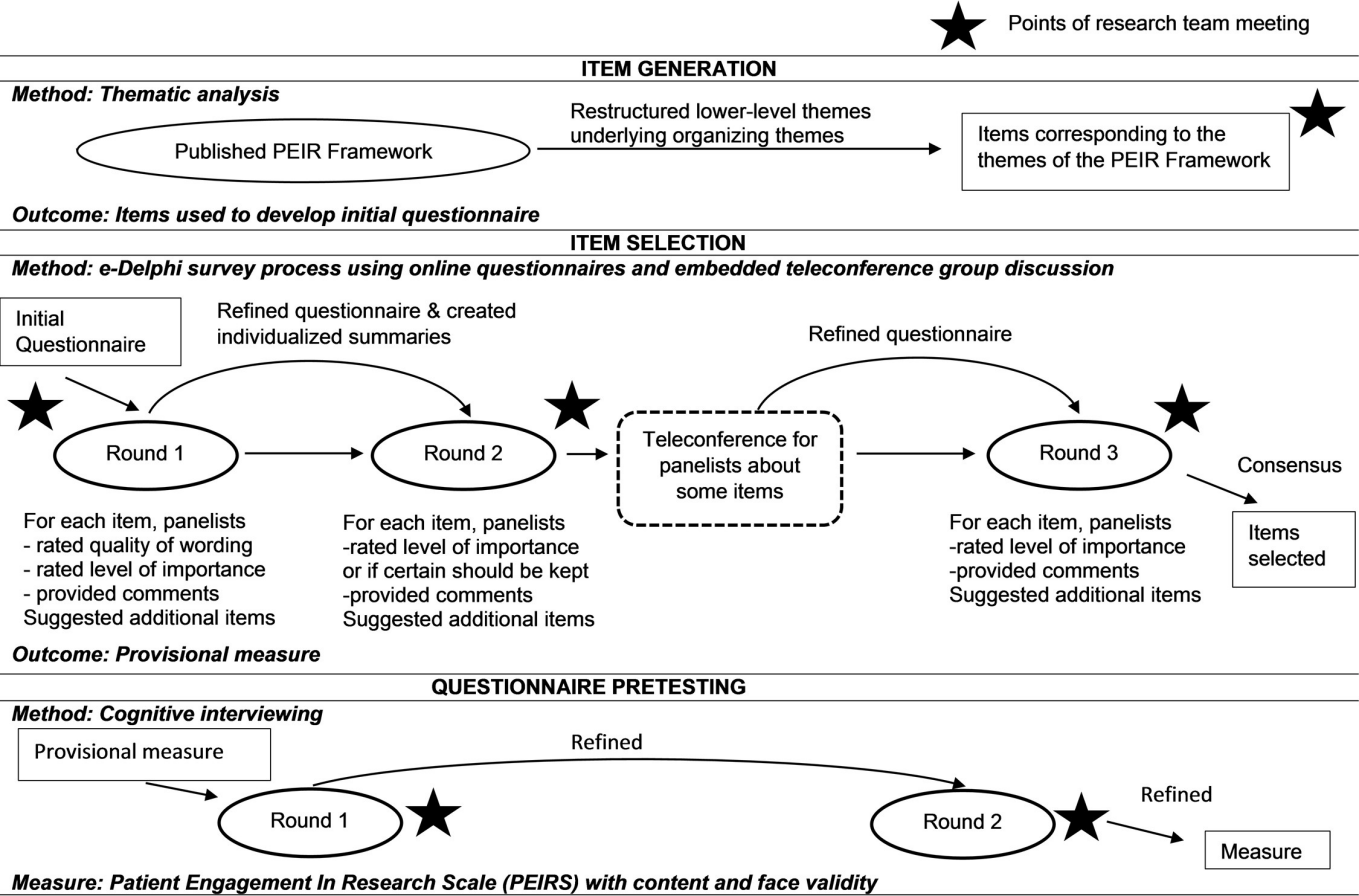
Three-phase study design for the development and pretesting of the questionnaire.

We used a mixed qualitative and quantitative study design. As depicted in Fig 1, our collaborative team of researchers (CBH, TA, NK, CLB, LCL) and four experienced patient partners (AMH, AMM, KE, SM) conducted this study in three phases: 1) item generation, 2) item selection, and 3) pretesting of the resulting questionnaire.

### Item generation

**Fig 2:**
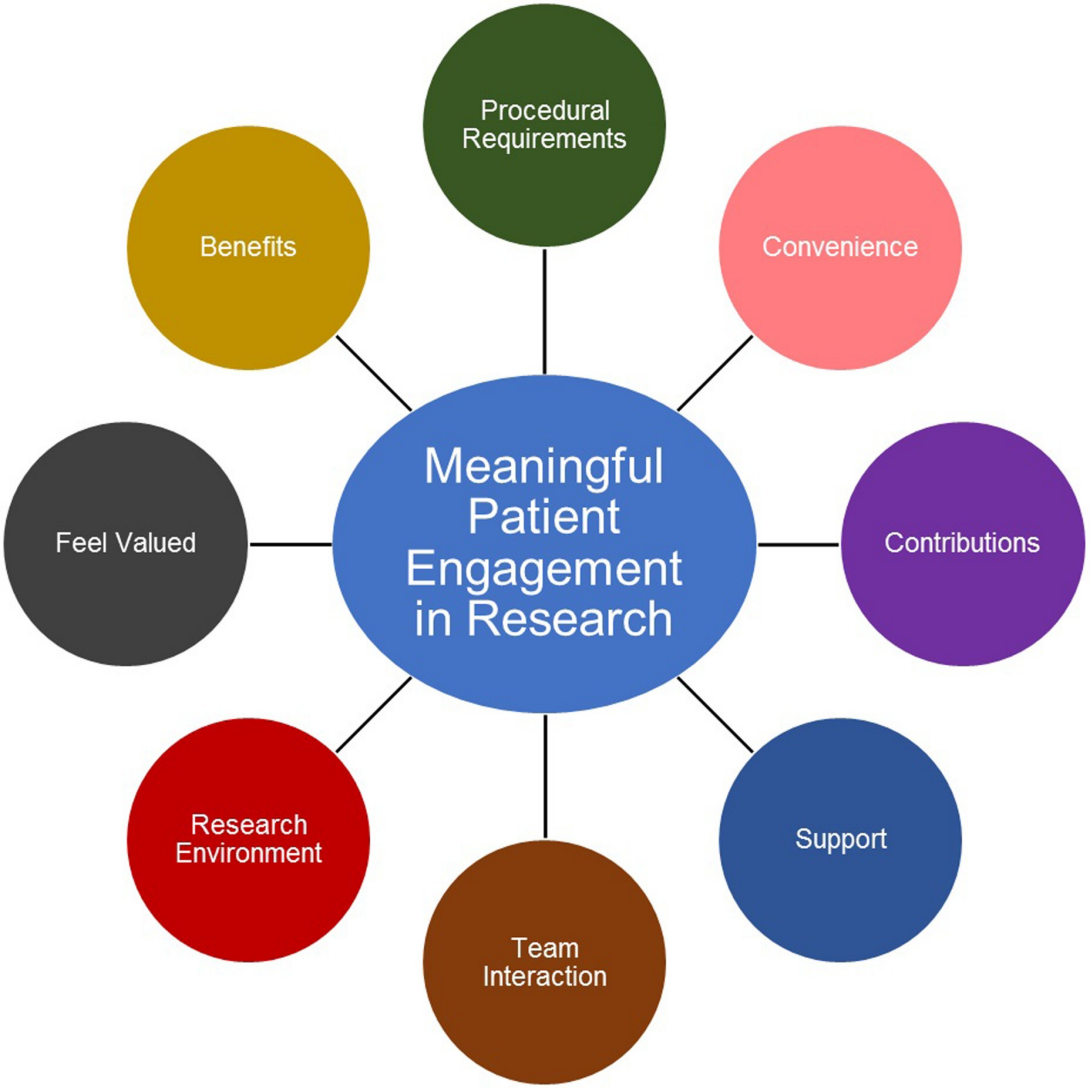
Patient Engagement In Research (PEIR) Framework. The PEIR Framework contains eight organizing themes that collectively define the meaningful engagement of patients in the research process from the perspective of patients.

Items were generated through a secondary qualitative analysis previously described for the development of the Patient Engagement In Research (PEIR) Framework (Fig 2).[6,35] The PEIR Framework has eight organizing themes for the elements of meaningful patient engagement in research projects from patients’ perspectives. Briefly, it was developed through thematic analysis of 18 transcripts of one-to-one in-depth interviews in a study exploring the *experiences and views of patients with arthritis* regarding their engagement in health research and supplemented by 18 publications related to public and patient engagement.[6,35] The lower-level themes underlying the eight organizing themes of the Framework were restructured into statements and used as the initial questionnaire items.

### Item selection

We conducted a three-round modified Delphi survey process via email correspondence to select and refine the questionnaire items (Fig 1).[36,37] The purpose of the e-Delphi survey was to build consensus among the target users about the items for the emergent measure. Teleconferences among participants were incorporated to enhance the participants’ assessment of items by providing a medium to discuss their positions on items.[38]

#### Settings and participants: e-Delphi survey

We sought to recruit a minimum of 10 participants.[36] Eligible individuals were adults (≥18 years old) who: 1) had engaged in research projects in Canada within the last three years and had reliable internet access; and 2) identified themselves as patients or informal caregivers, family members, or friends of patients. Recruitment was through email, websites, and social media accounts of health-related research networks or organizations and patient engagement organizations (e.g., Patient Voices Network) using a digital recruitment poster. We also recruited from our personal contacts and by word-of-mouth through study participants.

#### Data collection: e-Delphi survey

Before round one, our research team iteratively refined the initial questionnaire through discussions in a team meeting and subsequent email correspondence to remove redundancy and improve the comprehension and format of the questionnaire. The FluidSurveys (http://fluidsurveys.com) platform at the University of British Columbia hosted the questionnaire, and unique links were emailed to each participant. In round one, participants: 1) rated the quality of wording of each item (1–4; higher = better quality), 2) rated the level of importance (1–4; higher = more important), 3) provided alternative wording for the items (optional), and 4) suggested additional items (optional). The questionnaire was revised based on the results. In round two, individuals reviewed a personalized anonymized summary of their ratings compared against those of all other participants, and they then repeated steps 2 to 4 with the revised questionnaire. In addition, they reviewed items that did not meet all of the selection criteria (see below) and indicated which should be kept and which should be removed. Steps 2 to 4 were repeated in round three.

Between rounds two and three, each participant took part in one of two teleconference discussions co-facilitated by CBH and TA. Participants discussed certain items, chosen by our team, that had only partially passed the selection criteria. The facilitators took notes on suggestions for refining or removing each item.

#### Data analysis: e-Delphi survey

Across the three rounds, we applied two quantitative selection criteria to each item: 1) a median rating of ≥3.25 for level of importance, and 2) a rating of 3 or higher for level of importance by ≥70% of the e-Delphi survey participants.[39] We had a supplementary third criterion, the comments on an item’s wording and importance, when available. An item passed the selection criteria when both 1 and 2 were met, partially passed when only either 1 or 2 was met, and failed when neither was met. These criteria guided our decision to retain an item when it passed; revise when it passed, partially passed, or failed; or remove when it partially passed or failed. The supplementary criterion and ratings on the quality of wording in round one informed the revision of an item. In round two, to be selected, >50% of participants needed to respond ‘Yes’ to keeping certain items that partially passed round one.

Two researchers (CBH and TA) analysed and interpreted round one data to revise the questionnaire. After rounds two and three, the respective data were analysed and interpreted by our patient-researcher partnership team members (except CLB and NK) in a two-hour team meeting. Team members received a data summary one to two weeks before the meeting. CBH presented the summary of the data for each item during the meeting and made the agreed-upon recommended changes. Subsequently, the questionnaire was iteratively refined through email and in-person communication between CBH and individual team members. Data from round two and the teleconferences were combined to refine the questionnaire for round three. Finally, our team used round three data to build consensus for the items to include in the provisional measure. All calculations were performed using SAS software package, version 9.5 (Cary, North Carolina, USA).

### Pretesting of questionnaire

Using the provisional measure, we conducted two rounds of cognitive interviewing to proactively identify and fix potential problems that would contribute to measurement error in order to establish the face and content validity.[40] Verbal information from respondents completing the provisional measure was used to evaluate the quality and respondents’ interpretation of each item.[41] Our approach was guided by the Cognitive Interviewing Reporting Framework, which provides direction on high-quality reporting.[42]

#### Settings and participants: Cognitive interviewing

Cognitive interviewing has no agreed-upon minimum sample size,[40,43] but the number of participants typically varies between 5 and 15 per round.[41] Eligibility was consistent with the item selection phase, except that internet access was not required and individuals had to be available for an in-person interview in or near Vancouver, British Columbia. The recruitment strategy mirrored the item selection phase. Additionally, we contacted patient partners on email lists from research conferences and contacted participatory research teams.

#### Data collection: Cognitive interviewing

We constructed an interview guide (see S1 File). Three authors (CBH, AMM, LCL) used the Question Appraisal System (QAS-99) to independently review each item to identify possible problems to ask about.[40,44] QAS-99 is an eight-step checklist for identifying and fixing problems in questionnaire items.[40,44] The main probes were organized into five categories. Four were common cognitive processing problems for questionnaires (comprehension, information retrieval, judgment/estimation, reporting) as articulated in the Cognitive Aspects of Survey Methodology (CASM) model and one involved logical/structural problems.[45,46]

All interviews were scheduled for one hour at a mutually convenient location. In one-to-one interviews by ‘concurrent verbal probing,’ CBH asked each participant about an item immediately after or when responding to it, and about the instructions, structure, and usefulness of the questionnaire.[40] The interviewer attempted to selectively probe at least two participants’ views on each item.[40] Interviews were audio-recorded and transcribed verbatim for analysis. CBH has experience in psychometric evaluation of patient-reported measures and has published on the engagement of patients in research. He was trained by a senior qualitative researcher (CLB) on interview skills for this study.

#### Data analysis: Cognitive interviewing

After each round, two researchers (CBH and NK) segmented and reduced the interview data to match corresponding items and questionnaire instructions. They then coded for potential questionnaire problems. A list of the items and corresponding potential problems was emailed to the research team. Subsequently, the research team met, discussed, and agreed by consensus on a solution to keep, modify, or remove each potentially defective item. The changes were made by CBH, and further feedback requested from the team by email. Our collaborative approach sought to ensure the issues addressed were real rather than errors arising from using a researcher perspective exclusively.[41] Problems were addressed based on their logical merit as decided by our research team, rather than on their frequency. When a problem identified was applicable to other items, we made the appropriate changes. The data analysis was performed using NVivo software (version 11, QSR International Pty Ltd, Burlington, MA).

### Ethical considerations

Each participant in the item generation phase gave consent. Participants were offered an honorarium of $60 for the item selection and $40 for the pretesting phase. They gave informed written consent and indicated whether they wanted to be explicitly acknowledged in this paper. This study was approved by the Behavioural Research Ethics Board at the University of British Columbia (H16-02337).

### Overview of patient engagement

This was a researcher-initiated study.[21] Our collaborative research team included four patient partners, all Caucasian women with an arthritis diagnosis and previous experience of engaging in health research. They were members of the Arthritis Patient Advisory Board of Arthritis Research Canada. Patient partners were engaged from the preparatory phase (refining the research question and grant application) through to the ongoing translation phase. They contributed by reviewing and commenting on study documents through email, research team meeting discussions (whether in-person or remotely via teleconference and videoconference), recruitment of participants, presentation of study findings at conferences, and writing of this manuscript. Patient partners on our research team were vital for the stage of interpreting the participants’ comments. They contributed to subjective modifications of the questionnaire that reflected on the perspectives of the study participants, to reach our final decision on each item and the overall questionnaire.

## Results

### Item generation

We created 120 items. The details of the sample used for item generation are published elsewhere.[6] Notably, of the 18 participants, 17 were women, all were diagnosed with arthritis, and 12 had concurrent health conditions/diseases. These 120 items were divided into eight themes[38]: *Procedural Requirements* (n = 43), *Convenience* (n = 9), *Contributions* (n = 16), *Research Environment* (n = 5), *Team Interaction* (n = 12), *Support* (n = 7), *Feel Valued* (n = 12), and *Benefits* (n = 16).

### Item selection

Table 1 presents the demographic characteristics of the 12 participants in the e-Delphi survey. Most participants were women (83%), Caucasian (92%), and aged over 45 years (75%). They represented a variety of diseases, health-related conditions, and use of healthcare services. Their highest formal education ranged from high school diploma (n = 1) to master’s degree (n = 2). Two participants were recruited from the authors’ personal contacts. All participants completed each round, except one participant who missed round two because of an environmental disaster.

**Table 1:** Demographics characteristics of participants in the items selection and pretesting of the questionnaire.

Characteristics	Item Selection (n = 12)	Pretesting (n = 12)
Age (years)		
18–25	1	1
26–35	-	1
36–45	2	2
46–55	3	2
56–65	3	2
66–75	3	4
75–85	-	-
86+	-	-
Gender		
Women	10	3
Men	2	9
Race		
Indigenous populations	-	1
Asian	1	-
Black/African descent	-	1
Caucasian	11	10
Education		
High school diploma	1	-
Some college	3	3
College or trade school diploma	3	4
Bachelor’s degree	3	4
Master’s Degree	2	1
Doctoral degree or above	-	-
Role when engaged in research		
Research team member	7	10
Advisor to research team	11	9
Advisor on research priorities	7	8
Grant reviewer	7	7
Type of patient partner		
Patient	12	12
Family member of patient	5	3
Friend of patient	4	1
Informal caregiver	1	3
Country in which engagement took place		
Canada only	12	10
Canada and other	-	2
Additional language(s) spoken		
French	1	-
Cantonese	1	-
Time engaged in research		
Less than 1 year		1
1–3 years		5
3+ years		5
Grant reviewer		
Less than 1 year		7
1–3 years		1
3+ years		-
Advisory board member		
Less than 1 year		1
1–3 years		4
3+ years		3
Diseases, health-related conditions, and use of healthcare services	Rheumatoid arthritis, inflammatory bowel syndrome, multiple sclerosis, diabetes, stroke, neurodevelopmental disabilities, obesity, and nutrition intervention implementation.	Alzheimer’s disease, bursitis, cancer, cerebral palsy, Crohn’s disease, diabetes, multiple sclerosis, rheumatoid arthritis, alcohol abuse disorder, compression fracture, depression/anxiety, fragility, hearing loss, hepatitis C, HIV, leg amputation, osteoporosis, spinal cord injury, stroke, vertigo, “alcoholic/addict in early recovery”, care failure resulting in death, and lung transplant.

The questionnaire had 120 items in round one, 120 items in round two (86 rated for level of importance and 34 rated on whether or not they should be kept), and 57 items in round three. Table 2 shows distributions of the items with respect to meeting the two quantitative selection criteria within each round. During the two teleconferences, in July 2017, eight participants discussed four items in the first teleconference, and four participants discussed 11 items in the second.

**Table 2:** Summary of item selection through the e-Delphi survey process.

Theme	Round 1 (Total items = 120)			Round 2 (Total items = 120)			Round 3 (Total items = 57)		
	Passed	Partial	Failed	Passed	Partial	Failed	Passed	Partial	Failed
Procedural Requirements	24	12	7	17	14	11	12	8	2
Convenience	3	5	1	5	1	3	2	2	1
Contributions	9	4	3	4	6	6	4	1	-
Support	4	2	1	3	2	2	5	-	-
Research Environment	5	-	-	2	3	-	2	1	1
Team Interaction	3	7	2	3	4	5	3	-	1
Feel Valued	9	3	-	3	7	2	4	1	-
Benefits	8	6	2	2	9	5	2	3	2
TOTAL	65	39	16	39	46	34	34	16	7
SELECTED^a^	65	39	16	38	18	1	34	9	0

Passed: BOTH received a median rating of >3.25 AND rating of 3 or higher by ≥70% of participants. Partially passed: EITHER received a median rating of >3.25 OR rating of 3 or higher by ≥70% of participants. Failed: NEITHER received a median rating of >3.25 NOR rating of 3 or higher by >70% of participants.

^*a*^ Guided by selection criteria, but determined through research team discussions which considered participants comments about each item.

In round one of the e-Delphi survey, which took three and a half weeks, 65 items (54%) passed the quantitative selection criteria. Only four items were missing one rating each for level of importance. For quality of wording, most items had one to three missing responses. The majority of the items (n = 87) had a median rating of 3 (‘good’) for quality of wording, while 30 had a median rating of >3, and four had a median rating of <3. Fifty items were revised, and all 120 items were included in the refined questionnaire for round two.

In round two, which took four weeks, 38 items (32%) passed the quantitative selection criteria plus one item that >50% participants rated should be kept. (See S1 Appendix for a per item summary.) An example of an item discussed is “The project matched my interests,” which had a median of 3 and 91% of participants rated it ≥3 for level of importance. During the first teleconference, the word ‘suited’ was suggested to replace ‘matched,’ but ‘piqued’ was later suggested in the second teleconference. The item was modified accordingly, but eventually removed after the final round after it failed the selection criteria. A second example, the item “I was paid for my contributions,” had a median of 4, but only 64% of participants rated it as ≥3 (moderately or extremely important). This item was extensively discussed during the teleconferences. The first teleconference resulted in “I received sufficient payment for my contributions (…),” which was changed to “I was offered sufficient payment for my contributions (…)” during the second teleconference. It was important to include the word ‘offered’ because, for various reasons, some patient partners would not accept compensation. A total of 37 items were modified, 23 of which had passed the quantitative selection criteria.

Of the 57 items in round three, which took three weeks, 34 passed and nine partially passed the two quantitative selection criteria (see S2 Appendix). Twenty-three items were subsequently modified. The remaining 43 items were divided across the eight themes, from three items each for *Research Environment* and *Team Interaction* to 16 items for *Procedural Requirements*.

### Pretesting of questionnaire

Cognitive interviewing included 12 participants (round one: n = 5; round two: n = 7) between January and April 2018 (Table 1). One man had participated in the e-Delphi survey. Most participants were men (75%), Caucasian (83%), and aged over 45 years (67%). All identified as patients, and three also identified as both family members and informal caregivers. They reported having a variety of diseases or health-related conditions. Their highest formal education ranged from some college (n = 3) to master’s degree (n = 1). One participant was recruited from the authors’ personal contacts.

The cognitive interviews lasted between 24 and 77 minutes. In round one, each item had comments from 2 to 5 participants, except for two items that had one and no comments each. In round two, each item had comments from 2 to 7 participants.

Potential problems were identified in 32 items, and the general and theme-specific instructions (Table 3). Five of these items were addressed because of a potential problem identified within one item during round two. We identified 27 potential problems in round one and 32 in round two. We applied the CASM model and logical problems scheme 54 times across the items: comprehension (n = 26), retrieval (n = 2), judgment (n = 13), reporting (n = 4), and logical (n = 9). Four items were removed after round one and one more after round two. Four of the six items removed were too similar to other items, and two of them were integrated with other items. The fifth item, “I had the option of joining meetings remotely,” was subsequently removed after it was deemed by our team to not be broadly applicable, and the sixth item was considered redundant.

**Table 3:** Summary of results from the pretesting of the questionnaire.

Initial item	Item’s median score	Number of missing scores	Number of problems per round	Examples of potential problem	CASM category	Final decision
General instructions	N/A	N/A	0 2 |	- Participant had to re-read first statement, and would have re-worded it - Suggestion to include approximate completion time	Logical	Modified to include approximate completion time
Instruction for each category of items	N/A	N/A	0 | 0	Timeline were not clear for some items	Logical	Instructions modified by adding “throughout the project”
The number of patient partners on the research team was appropriate	3	0	1 | 2 |	- Not sure about correct number, but selection and reason consistent with intent of item ————————————————— - Recall failure: Felt number was appropriate, but was unable to retrieve number - Sensitive wording: The word “patient” was not well liked, but participants could not provide an alternative	Judgment ———- Retrieval Comprehension	The number of patient partners on the research project team seemed appropriate
I had a clear understanding of my role	3	0	2 | N/A	- Too similar to another item - Reference period: Interpreted only for the beginning of the project	Logical Comprehension	Removed
I understood the research goals	3	0	0 1 |	- Technical term: Too similar to another item, “goal” and “objective” viewed as interchangeable	Comprehension	I understood the objective(s) of the project
I agreed with the purposes of the project	4	0	1 | 1	- Technical term: View as a question for qualified people, and contemplated use of “purpose” vs “goal” —————————————— - Too similar to another item, ‘goal’ and ‘objective’ viewed as interchangeable	Judgment Comprehension —— Logical	I agreed with the objective(s) of the project
I understood the expectations for my contributions to the project	4	0	2 | 1	- Too similar to another item - Wording: Suggested “I understood what I was supposed to do” —————————————— - Viewed as already addressed by previous items	Logical, Comprehension —- Logical	I understood how I could contribute to the project
I understood my ethical responsibilities for the project	3	0	1 1	- Vague: Two different, but credible interpretations —————————————— - Spoke about respect rather than ethical responsibility	Comprehension — Comprehension	Keep
I had sufficient opportunities to contribute to the project	3	0	1 | 0	- Reference period: Viewed as dependent on reference point throughout project	Judgment	In general, I had sufficient opportunities to contribute to the project
Communication within the research team was clear	3.5	0	0 1 |	- Self is not within research team, but an advisory board member	Judgment	Communication within the research project team was clear throughout the project
Throughout the project, there was feedback between me and the research team	3	0	0 1 |	- Similar to item about sufficient update, feedback vs communication	Logical	Removed and integrated into above item
The project was worth the time I spent on it	3.5	0	0 1	- Suggested expanding regarding compensation, but recognized this is addressed in a later question	Comprehension	Keep
I could choose my tasks in the project	3	0	2 | 3 |	- Understood but found it hard to answer - Disagree but found level of engagement appropriate —————————————— - Wording: Viewed wording as confusing - Knowledge: Cannot respond at onset of project when workload is unknown - Knowledge: Hard to answer, wasn’t until after first meeting they had input - Did not understand because team selected them, not advisory board	Reporting —- Comprehension Judgment	I had the opportunity to provide input into selecting my tasks for the project
I had sufficient time to complete my tasks for the project	3	0	1 2 |	- Reference period: Whether to include all tasks up to completing the questionnaire —————————————— - Considered putting neutral because the project was not completed yet - Vague: Which perspective, “I had the time or the team provided the time”?	Judgment — Judgment Comprehension	Throughout the project, I had sufficient time to complete my tasks for the project
I contributed by providing my perspective as a research partner	4	0	1 | 0	- Technical term: Not sure about relevance, suggested removal of “research partner”	Comprehension	I contributed by providing my perspective
My contributions were a good use of my time	3.5	0	1 | 0	- Mismatch: Understood question but did not select response	Reporting	Keep
I shared my knowledge within the team	3.5	0	0 1 |	- Self is not within research team, but an advisory board member	Comprehension	I shared my knowledge within the project team
Throughout the project, I felt accepted as a member of the research team	4	0	0 1 |	- Self is not within research team, but an advisory board member	Comprehension	Throughout the project, I felt accepted as a member of the research project team
I was an equal partner in the research team	3	0	0 2 |	- Self is not within research team, but an advisory board member - Does not need to be equal with researcher, felt valued but not equal	Comprehension Judgment	I was an equal partner in the research project team
I had the option of joining meetings remotely	4	0	1 | N/A	- Inappropriate assumption: Item considered irrelevant to their project	Comprehension	Removed
My interactions within the research team were positive	4	0	0 1 |	- Self is not within “research team”, but an advisory board member - Technical term: Understood, but “interaction” might not be best word for average person	Comprehension	My interactions within the research project team were positive
There was mutual respect among the research team members	4	0	0 1 |	- Self is not within “research team”, but an advisory board member	Comprehension	There was mutual respect among the research project team members
There was trust among the research team members	3.5	0	0 1 |	- Self is not within research team, but an advisory board member	Comprehension	There was trust among the research project team members
I received sufficient support to contribute to the project	4	0	2 | 0	- Selected neutral because they did not need training - Initially, did not know how to respond	Judgment, Comprehension	I received sufficient reimbursement for out-of-pocket expenses (such as childcare, parking, travel) related to the project activities
I received the training I needed for my role	3	0	2 | N/A	- Selected neutral because they did not need training - Initially, did not know how to respond	Reporting	Removed and integrated into above item
My concerns were addressed	3	1	0 4 |	- Vague: Wanted clarification on which concerns - Item was too broad - Trouble remembering, but did describe scenarios in which his concerns were addressed - Inappropriate assumption: Assumes there were concerns	Comprehension Retrieval	Any concerns I had were addressed
I had access to both financial and non-financial resources for my involvement in the project	1	1	1 | N/A	- Item is double-barreled	Reporting	Removed and integrated into above question
I was offered sufficient reimbursement for my out-of-pocket expenses (such as childcare, parking, and travel) related to project activities	3	1	2 | 0	- Interpreted reimbursement as compensation - Not applicable to their projects	Comprehension Judgment	Keep
The research team appreciated my contributions	4	0	1 1 |	- Too similar to next item; “appreciated” vs “valued” —————————————— - Self is not within research team, but an advisory board member	Logical ——- Comprehension	The research project team appreciated my contributions
The research team valued my contributions	3	0	1 | N/A	- Viewed as too similar to previous item; “appreciated” vs “valued”	Logical	Removed
The research team was open to receiving my views	4	0	0 2	- Uncertain about wording because quiet patient partner might not contribute - Self is not within research team, but an advisory board member	Judgment Comprehension	The research project team was open to receiving my views
I was offered sufficient compensation for my contributions	3.5	2	3 | 1	- Viewed compensation as monetary - Viewed as not applicable, because did not get payment - Did not get payment, but thinks getting it would not hurt —————————————— - Wording: Viewed “compensation” as general financial attachment but networking suggested otherwise	Judgment — Comprehension	I was offered sufficient recognition for my contributions (for example, payment, authorship, or gifts)
I saw how my contributions could benefit other people	4	0	1 | 0	- Vague: Asked if benefit was for research team or public	Comprehension	I saw how my contributions could benefit others
My involvement had positive impacts on my life	4	0	0 1	- Technical term: Initially, suggested “impact” means a direct impact; there is no impact, but felt positive about own involvement	Comprehension Judgment	Keep

Time of revision indicated by vertical line |. Potential problems divided by round indicated by horizontal dash line ——. CASM, Cognitive Aspects of Survey Methodology. PEIRS Scores: 4 –Strongly Agree; 3 –Agree; 2 –Neutral; 1 –Disagree; 0 –Strongly Disagree.

One participant’s recommendation, subsequently affirmed by other participants, informed the modification of the general instruction section of the measure to include information about its expected completion time. The theme-specific instructions were modified to clearly indicate that respondents should consider their entire experience throughout a research project when responding to each item.

In round one, the number of themes in the PEIRS was reduced from eight to seven after *Research Environment* and *Team Interaction* were combined into *Team Environment and Interaction*. The original themes had overlapping constructs, and our team decided that the small number of items could be represented by a single theme. Across the two rounds, the item “I was offered sufficient compensation for my contributions (…),” in the *Feel Valued* theme, stood out as a contentious item. Participants had diverse opinions about its inclusion. In round two, one participant noted that the term ‘compensation’ often has a financial connotation, and suggested that the word ‘recognition’ be included in parentheses to circumvent potential ambiguity. Our research team replaced the word ‘compensation’ with ‘recognition,’ noting that it was a more comprehensive description of the ways in which patient partners are shown appreciation for their contributions to a research project. CBH observed no differences in views on items by gender or type of patient partner.

### Patient Engagement In Research Scale (PEIRS)

The resulting measure, the Patient Engagement In Research Scale (PEIRS), contains 37 items distributed across seven themes of meaningful engagement of patients in research that could potentially operate as subscales (see S2 File). There are three to five items in six of the themes, and 14 in the *Procedural Requirements* theme. The PEIRS currently uses a five-point Likert scale to rate each item, and likely takes 10 to 15 minutes to be completed. Numbers were not added within the response categories of the Likert scale, because labelling the Likert scale with numbers rather than adjectives might entail different cognitive processing (for example, 4/5 versus Agree/Strongly Agree) when a patient partner is estimating their experiences as captured by each item. Total scores generated by the PEIRS will be interpretable only after its measurement properties have been determined in a subsequent study. (See S3 File for a guide to calculating the total scores.) Finally, the PEIRS achieved a Flesch Reading Ease score of 71.2 in Microsoft Word 2016, demonstrating it is suitable for reading at a 7^th^-grade level or higher.

## Discussion

There is increased utilization of participatory research approaches that include patient partners on health research project teams.[47] This study developed and pretested a self-administered questionnaire for patient partners to self-report the quality of their engagement in research projects. To our knowledge, the PEIRS is the first measure developed to assess the quality of patient engagement in research in a comprehensive way,[48] and first to be built primarily from the perspectives of patient partners. This study is, in part, a response to the reported need for such a measure to determine effective methods for engaging patients in research.[3,15,32] Instead of focusing on the dimensions of process, context, and impact outline by Esmail et al.,[32] we had a broader focus on the quality of engagement. Meaningful engagement as the construct underlying the quality of engagement encapsulates aspects of those dimensions.[6] A main strength of this study is our detailed approach to ensure the PEIRS was grounded on the experiences and views of patients (inclusive of their informal caregivers, family members, and friends) who engage in research project teams.

Both the e-Delphi survey process and cognitive interviewing process (i.e., cognitive testing) provided content validation for the generated items. This demonstrated that the participants viewed the items as comprehensible and acceptable for capturing degrees of meaningful patient engagement in research.[36,49] Through the e-Delphi survey, we determined the highly important items for capturing meaningful engagement. Both processes endorsed the items’ placements within the themes of the PEIR Framework. No additional items were added during the e-Delphi survey, which suggests the PEIRS is a comprehensive way to capture what patient partners value as the essential elements of meaningful engagement in research. The e-Delphi survey built anonymous consensus among the study participants using their independent ratings, while limiting any bias arising from the influence of any participant.[36,38] The teleconferences would not have affected the anonymity of the responses because participants responded independently and were blinded to each other’s responses during each round of the e-Delphi survey. Thus, the process makes the case that the elements of meaningful engagement were legitimately shared across individuals with varied experiences as patient partners.

In addition to content validation, the cognitive interviewing process helped to establish face validity of the PEIRS. This demonstrated that participants subjectively endorsed the PEIRS as appropriate for capturing meaningful engagement of patients in research.[49] Through the interviews, participants affirmed the acceptability of the items included in the PEIRS. Furthermore, we corrected potential problems in the questionnaire that would ensure its content is clearly presented and understandable, and has no ambiguity or redundancies. Theoretically, addressing those problems mitigates potential measurement errors in the scores that will be generated by the PEIRS.

This study has limitations. The sample of the e-Delphi survey consisted mainly of English-speaking Caucasian women. The views of other ethnic groups and gender identities might have been inadequately reflected in the provisional PEIRS. The cognitive interviewing increased the contribution of the perspectives of men and non-Caucasian ethnicities to the content of the PEIRS. Overall, the ideas within each item of the PEIRS were endorsed by participants with varied experiences as patient partners and with a variety of ethnic, gender, and other demographic characteristics. Finally, our research team determined the final content of the PEIRS, rather than performing cognitive interviews until no additional potential problems were identified. The inclusion of experienced patient partners on our team made this process credible, although the fact that they were all women with arthritis could be perceived as a limitation.

### Context and interpretation

The construct underlying the PEIRS is deemed multidimensional and accounts for experiences throughout the entirety of a patient-researcher partnership.[6] The PEIRS is designed for an adult patient partner to complete about their own perspective of being engaged in a research project. Its readability meets recently published criteria,[48] which demonstrates it appropriate even for individuals will low degrees of reading skills in the English-language. Its administration would be appropriate after a research project team has had sufficient activities for a patient partner to have experiences to reflect upon. Individuals could complete it at multiple points throughout the life cycle of a research project. The PEIRS is designed to test patient engagement methods/interventions in cross-sectional and longitudinal analyses. Finally, it is intended for both individual- and group-level evaluations.

### Future directions

The PEIRS is currently undergoing psychometric testing to establish its measurement properties for descriptive and evaluative applications. This follow-up online survey study seeks to establish reliability/reproducibility, construct validity, and interpretability of the scores generated by the PEIRS within a broad range of adult patient partners in different age categories, diseases/conditions and healthcare services, and locations across Canada.[49] This could lead to further modification of the PEIRS. A subsequent study should investigate the responsiveness of the validated PEIRS.[49] Individual-level assessments, as opposed to group-level, might be needed when monitoring and evaluating research projects that engage few patient partners.

Future studies could conduct cross-cultural adaptation of the PEIRS. Studies could also investigate the validity of the PEIRS for use with children who are patients, the public in general, or specific populations, such as Indigenous Peoples, who engage on health research teams.

## Conclusions

We developed a 37-item self-reported questionnaire, the Patient Engagement In Research Scale (PEIRS), for quantifying meaningful patient engagement in research for the evaluation of the quality of patient engagement. The PEIRS is grounded in the perspectives of patient partners, who provided preliminary content and face validity. The patient partners on our research team helped to ensure that the subjective refinement of the PEIRS reflected a patient partner perspective. The measurement properties of the PEIRS still need to be studied and demonstrated.

## Supporting information

S1 File Cognitive interviewing guide. (DOCX)

S2 File Patient Engagement In Research Scale (PEIRS). (PDF)

S3 File Scoring guide for the Patient Engagement In Research Scale (PEIRS). (DOCX)

S1 Appendix Per-item summary of the rating of level of importance in e-Delphi round two. (DOCX)

S2 Appendix Per-item summary of the rating of level of importance in e-Delphi round three. (DOCX)
