# Supplementary material for: Development and pre-testing of the Patient Engagement In Research Scale (PEIRS) to assess the quality of engagement from a patient perspective
Source: PLoS One. 2018 Nov 1;13(11):e0206588. doi: 10.1371/journal.pone.0206588 (PMC6211727; doi:10.1371/journal.pone.0206588)
Supplement: S1 File — (DOCX) [file pone.0206588.s001.docx]

**Cognitive Interviewing Guide**

**Getting Started**

- Thank participant for deciding to participant in the study.
- Start with a bit of chit-chat to build rapport, while being respectful of the participant’s time, but without rushing.

**Consent**

- Re-iterate the purpose of the study and refresh the participant on the details in consent form.
- Ask the participant, “Do you have any questions before we begin?”

**Interrupting sentences**

- Before you move on to the next statement …
- Let’s pause, when you were looking at statement **___** …

**Providing feedback:**

- - Thank you, that’s helpful.
  - This is helpful (useful) to our study.
  - All of this is useful for the study.
  - All the information is helpful.

** *Instruction to self*: Do not provide personal feedback.

**Cognitive Interviewing Guide for Verbal Probing**

**Instructions to Interviewee**

The purpose of our session today is to hear your thoughts about the PEIRS for its use to assess meaningful patient engagement in research. So this interview today is to collect information for refining the PEIRS, and is not specifically about you. Thank you again for consenting to participate in this study and for allowing us to audio-record it.

Do you have any questions before we begin?

I am going to keep interrupting you to ask what you are thinking as you complete the questionnaire. I will want to know if some statements in the PEIRS are ***difficult to understand***, ***hard to answer***, or ***make little sense***.

We are mainly interested in the ways that our participants arrive at their responses to the statements, and the problems they encountered while responding. During this interview, we are interested in any detailed help you can give, even if it might seem irrelevant.

*****I will now turn on the recorder. [Say Time, Date, Location, and Participant]**

Here is the PEIRS questionnaire [Hand questionnaire to participant]. I would like you to start by filling out the information at the top, then respond to each statement.

[PAUSE]

I will need to watch as you complete the questionnaire, because that is what the study is about.

**PROBES**

* Most probes were selected and adapted from the how-to-guide on cognitive interviewing by Gordon Willis.^[[1]](#footnote-1)^

**Comprehension**

These probes have to do with 1) what the participant thinks the statement is asking, and what the specific words and phrases in the statements mean to the participant.

1. What does the term “__________” mean to you?
2. Can you repeat the statement “read the PEIRS statement or statement code” in your own words?
3. What, to you, is ___________?

**Recall**

These probes have to do with 1) the type of information the participant needs to recall in order to respond to the statement, and 2) the type of strategy the participant relies on when retrieving information (for example, accurate recall or estimation).

1. How do you remember that ….?
2. How well do you remember this?
3. What period of time are you thinking of here, specifically?
4. Was this easy or hard to answer?
5. Was it difficult to respond to this/these statement/s?

**Judgment/Decision/Estimation/Bias**

In line with the concept of social desirability, these probes have to do with 1) does the respondent devote enough mental effort to respond accurately and thoughtfully to the statement?, and 2) does the participant want to tell the truth, or does the participant say something that sounds “better”?

1. What kinds of things were you thinking of when you were responding to this statement.
2. How sure are you of your response?
3. How sure are you that …. ?
4. Why do you think that ….?
5. How did you arrive at the response “participant’s response”?
6. I notice that you hesitated before you answered “PEIRS statement”, what were you thinking?
7. How hard was this to respond to?

**Response process**

1. Why did you select “participant’s response”?
2. Were the response options appropriate?

**Logical/Structure**

1. Did the PEIRS provide clear instructions?
2. In general, what do you think about the PEIRS questionnaire?

**Conditional Probes**

| Condition | Conditional Probe |
| --- | --- |
| 1. Participant cannot respond or does not know the response. | What was going through your mind as you tried to respond to the statement? |
| 1. Participant responds after a period of waiting. | You took a little while to response to that statement. What were you thinking about?  ***See Judgment #5** |
| 1. Participant responds with uncertainty, using explicit cues such as “um”, “ah,” changing a respond, etc. | You seem to be somewhat uncertain. If so, can you tell me why? … What caused you to change your response? |
| 1. Erroneous response; verbal report implies misconception or inappropriate response process. | Clarify participant’s understanding of the particular term or the process used. For example, if the participant appears to misunderstand the word “partner,” probe the term (“so you don’t consider yourself to be a partner? |
| 1. Participant requests information instead of providing a response. | If I weren’t available or able to answer, what would you decide it means? Are there different things you think it might mean? What sort of things? |

**Closing**

**Note to self: Don’t turn off the recorder because more conversation might happen. If participant asks, then turn it off.

**Thank you**

That concludes my questions, do you have anything that you would like to add? Thank you for your participation in the PEIRS study.

**Honorarium**

I would like to offer you $40 for your participation in this study.

1. Willis, G. B. (1999). Cognitive interviewing: A “how to” guide. Research Triangle Park, NC: Research Triangle Institute. [↑](#footnote-ref-1)
