# Supplementary material for: Development and pre-testing of the Patient Engagement In Research Scale (PEIRS) to assess the quality of engagement from a patient perspective
Source: PLoS One. 2018 Nov 1;13(11):e0206588. doi: 10.1371/journal.pone.0206588 (PMC6211727; doi:10.1371/journal.pone.0206588)
Supplement: S2 File — (PDF) [file pone.0206588.s004.pdf]

PEIRS

Patient Engagement In Research Scale - PEIRS

Name: ______________________________

Date: ______________________________

Your project's name: ______________________________

____

INSTRUCTIONS: Thinking about your experience as a patient partner in the project, please respond to the statements by ticking only one box for each statement. If you are unsure about which option to choose for a statement, please give the best response you can. This questionnaire may take you about 10 to 15 minutes to complete.

Procedural Requirements

The following fourteen (14) statements are about your general experiences throughout the project.

PR1. I was interested in the issue(s) being researched in the project

Strongly Agree	Agree	Neutral	Disagree	Strongly Disagree
☐	☐	☐	☐	☐

PR2. The research team members were properly introduced to each other

Strongly Agree	Agree	Neutral	Disagree	Strongly Disagree
☐	☐	☐	☐	☐

PR3. The number of patient partners on the research project team seemed appropriate

Strongly Agree	Agree	Neutral	Disagree	Strongly Disagree
☐	☐	☐	☐	☐

PR4. I understood the objective(s) of the project

Strongly Agree	Agree	Neutral	Disagree	Strongly Disagree
☐	☐	☐	☐	☐

PR5. I agreed with the objective(s) of the project

Strongly Agree	Agree	Neutral	Disagree	Strongly Disagree
☐	☐	☐	☐	☐

PEIRS © 2018 C. Hamilton et al, All rights reserved

PR6. I understood how I could contribute to the project

Strongly Agree	Agree	Neutral	Disagree	Strongly Disagree
☐	☐	☐	☐	☐

PR7. I received sufficient explanation about the project

Strongly Agree	Agree	Neutral	Disagree	Strongly Disagree
☐	☐	☐	☐	☐

PR8. I understood my ethical responsibilities for the project

Strongly Agree	Agree	Neutral	Disagree	Strongly Disagree
☐	☐	☐	☐	☐

PR9. In general, I had sufficient opportunities to contribute to the project

Strongly Agree	Agree	Neutral	Disagree	Strongly Disagree
☐	☐	☐	☐	☐

PR10. I was able to perform my tasks for the project

Strongly Agree	Agree	Neutral	Disagree	Strongly Disagree
☐	☐	☐	☐	☐

PR11. I participated in making decisions about the project

Strongly Agree	Agree	Neutral	Disagree	Strongly Disagree
☐	☐	☐	☐	☐

PR12. I received sufficient updates about the project

Strongly Agree	Agree	Neutral	Disagree	Strongly Disagree
☐	☐	☐	☐	☐

PR13. Communication within the research team was clear throughout the project

Strongly Agree	Agree	Neutral	Disagree	Strongly Disagree
☐	☐	☐	☐	☐

PR14. The project was worth the time I spent on it

Strongly Agree	Agree	Neutral	Disagree	Strongly Disagree
☐	☐	☐	☐	☐

PEIRS © 2018 C. Hamilton et al, All rights reserved

Convenience

The following four (4) statements are about how convenient it was for you to contribute throughout the project.

CN1. I had the opportunity to provide input into selecting my tasks for the project

Strongly Agree	Agree	Neutral	Disagree	Strongly Disagree
☐	☐	☐	☐	☐

CN2. My preferences for meetings (such as time, duration, location, and format) were considered when planning meetings

Strongly Agree	Agree	Neutral	Disagree	Strongly Disagree
☐	☐	☐	☐	☐

CN3. Throughout the project, I had sufficient time to complete my tasks for the project

Strongly Agree	Agree	Neutral	Disagree	Strongly Disagree
☐	☐	☐	☐	☐

CN4. I had opportunities to express my views

Strongly Agree	Agree	Neutral	Disagree	Strongly Disagree
☐	☐	☐	☐	☐

Contributions

The following four (4) statements are about your contributions throughout the project.

CT1. I contributed by providing my perspective

Strongly Agree	Agree	Neutral	Disagree	Strongly Disagree
☐	☐	☐	☐	☐

CT2. My contributions were a good use of my time

Strongly Agree	Agree	Neutral	Disagree	Strongly Disagree
☐	☐	☐	☐	☐

CT3. I shared my knowledge within the project team

Strongly Agree	Agree	Neutral	Disagree	Strongly Disagree
☐	☐	☐	☐	☐

CT4. My workload in the project was manageable

Strongly Agree	Agree	Neutral	Disagree	Strongly Disagree
☐	☐	☐	☐	☐

PEIRS © 2018 C. Hamilton et al, All rights reserved

Team Environment and Interaction

The following five (5) statements are about the research environment and interaction throughout the project.

T1. Throughout the project, I felt accepted as a member of the research project team

Strongly Agree	Agree	Neutral	Disagree	Strongly Disagree
☐	☐	☐	☐	☐

T2. I was an equal partner in the research project team

Strongly Agree	Agree	Neutral	Disagree	Strongly Disagree
☐	☐	☐	☐	☐

T3. My interactions within the research project team were positive

Strongly Agree	Agree	Neutral	Disagree	Strongly Disagree
☐	☐	☐	☐	☐

T4. There was mutual respect among the research project team members

Strongly Agree	Agree	Neutral	Disagree	Strongly Disagree
☐	☐	☐	☐	☐

T5. There was trust among the research project team members

Strongly Agree	Agree	Neutral	Disagree	Strongly Disagree
☐	☐	☐	☐	☐

Support

The following three (3) statements are about the support provided throughout the project.

SU1. I received sufficient support to contribute to the project (for example, orientation, readings, training workshops, webinars)

Strongly Agree	Agree	Neutral	Disagree	Strongly Disagree
☐	☐	☐	☐	☐

SU2. Any concerns I had were addressed

Strongly Agree	Agree	Neutral	Disagree	Strongly Disagree
☐	☐	☐	☐	☐

PEIRS © 2018 C. Hamilton et al, All rights reserved

SU3. I was offered sufficient reimbursement for my out-of-pocket expenses (such as childcare, parking, and travel) related to the project activities

Strongly Agree	Agree	Neutral	Disagree	Strongly Disagree
☐	☐	☐	☐	☐

Feel Valued

The following three (3) statements are about your feeling of being a valued member of the research team.

FV1. The research project team appreciated my contributions

Strongly Agree	Agree	Neutral	Disagree	Strongly Disagree
☐	☐	☐	☐	☐

FV2. The research project team was open to receiving my views

Strongly Agree	Agree	Neutral	Disagree	Strongly Disagree
☐	☐	☐	☐	☐

FV3. I was offered sufficient recognition for my contributions (for example, payment, authorship, or gifts)

Strongly Agree	Agree	Neutral	Disagree	Strongly Disagree
☐	☐	☐	☐	☐

Benefits

The following four (4) statements are about the benefits of your involvement in the project.

BE1. I enjoyed being a part of the project

Strongly Agree	Agree	Neutral	Disagree	Strongly Disagree
☐	☐	☐	☐	☐

BE2. I made an impact on the decisions in the project

Strongly Agree	Agree	Neutral	Disagree	Strongly Disagree
☐	☐	☐	☐	☐

BE3. I saw how my contributions could benefit others

Strongly Agree	Agree	Neutral	Disagree	Strongly Disagree
☐	☐	☐	☐	☐

BE4. My involvement had positive impacts on my life

Strongly Agree	Agree	Neutral	Disagree	Strongly Disagree
☐	☐	☐	☐	☐

PEIRS © 2018 C. Hamilton et al, All rights reserved
