# Supplementary material for: Development and pre-testing of the Patient Engagement In Research Scale (PEIRS) to assess the quality of engagement from a patient perspective
Source: PLoS One. 2018 Nov 1;13(11):e0206588. doi: 10.1371/journal.pone.0206588 (PMC6211727; doi:10.1371/journal.pone.0206588)
Supplement: S3 File — (DOCX) [file pone.0206588.s005.docx]

**Name:** ____________________________________________________

**Example for six items**

| **Likert Scale** | **Score** | **Selected** | **Sum** |
| --- | --- | --- | --- |
| Strongly Agree | 4 | X 3 | 12 |
| Agree | 3 | X 2 | 6 |
| Neutral | 2 | X 1 | 2 |
| Disagree | 1 | X 0 | 0 |
| Strongly Disagree | 0 | X 0 | 0 |
| **Total =** | | | 20 |

**Procedural Requirements**

| **Likert Scale** | **Score** | **Selected** | **Sum** |
| --- | --- | --- | --- |
| Strongly Agree | 4 |  |  |
| Agree | 3 |  |  |
| Neutral | 2 |  |  |
| Disagree | 1 |  |  |
| Strongly Disagree | 0 |  |  |
| **Total =** | | |  |

**Contributions**

| **Likert Scale** | **Score** | **Selected** | **Sum** |
| --- | --- | --- | --- |
| Strongly Agree | 4 |  |  |
| Agree | 3 |  |  |
| Neutral | 2 |  |  |
| Disagree | 1 |  |  |
| Strongly Disagree | 0 |  |  |
| **Total =** | | |  |

**Support**

| **Likert Scale** | **Score** | **Selected** | **Sum** |
| --- | --- | --- | --- |
| Strongly Agree | 4 |  |  |
| Agree | 3 |  |  |
| Neutral | 2 |  |  |
| Disagree | 1 |  |  |
| Strongly Disagree | 0 |  |  |
| **Total =** | | |  |

**Benefits**

| **Likert Scale** | **Score** | **Selected** | **Sum** |
| --- | --- | --- | --- |
| Strongly Agree | 4 |  |  |
| Agree | 3 |  |  |
| Neutral | 2 |  |  |
| Disagree | 1 |  |  |
| Strongly Disagree | 0 |  |  |
| **Total =** | | |  |

**Convenience**

| **Likert Scale** | **Score** | **Selected** | **Sum** |
| --- | --- | --- | --- |
| Strongly Agree | 4 |  |  |
| Agree | 3 |  |  |
| Neutral | 2 |  |  |
| Disagree | 1 |  |  |
| Strongly Disagree | 0 |  |  |
| **Total =** | | |  |

**Team Environment and Interaction**

| **Likert Scale** | **Score** | **Selected** | **Sum** |
| --- | --- | --- | --- |
| Strongly Agree | 4 |  |  |
| Agree | 3 |  |  |
| Neutral | 2 |  |  |
| Disagree | 1 |  |  |
| Strongly Disagree | 0 |  |  |
| **Total =** | | |  |

**Feel Valued**

| **Likert Scale** | **Score** | **Selected** | **Sum** |
| --- | --- | --- | --- |
| Strongly Agree | 4 |  |  |
| Agree | 3 |  |  |
| Neutral | 2 |  |  |
| Disagree | 1 |  |  |
| Strongly Disagree | 0 |  |  |
| **Total =** | | |  |

**PEIRS Total Score** = ____________________

$\frac{Total sum of themes}{152}\times100=$PEIRS Total

$$\frac{}{152}\times100=$$
