# Supplementary material for: Development and pre-testing of the Patient Engagement In Research Scale (PEIRS) to assess the quality of engagement from a patient perspective
Source: PLoS One. 2018 Nov 1;13(11):e0206588. doi: 10.1371/journal.pone.0206588 (PMC6211727; doi:10.1371/journal.pone.0206588)
Supplement: S1 Appendix — (DOCX) [file pone.0206588.s002.docx]

**S1** **Appendix** Per-item summary of the ratings for level of importance in round two of the Delphi survey

The items are categorized based on the two quantitative selection criteria. A third, supplementary, selection criteria was participants’ comments. The final decision to select an item was based on our research team discussion and consensus.

**Items that met the selection criteria**

| **Code** | **Item** | **Median** | **% score 3 and 4** |
| --- | --- | --- | --- |
| **Procedural Requirements (n=16)** | |  |  |
| **PR3** | The project was worth the time I spent on it | 4 | 100 |
| **PR6** | I understood the ethical requirements for the research project | 4 | 100 |
| **PR7** | I was encouraged within the research team to contribute | 4 | 91 |
| **PR12** | My role and tasks fit with my skills | 4 | 100 |
| **PR13** | My role and tasks fit with my availability | 4 | 100 |
| **PR14** | Team members were introduced to each other | 4 | 91 |
| **PR18** | I was involved in making decisions about the project | 4 | 82 |
| **PR21** | There was an appropriate number of patient partners | 4 | 100 |
| **PR22** | I had a clear understanding of my role | 4 | 100 |
| **PR23** | I had a clear understanding of my expected contributions | 4 | 100 |
| **PR26** | I was interested in the issue being researched | 4 | 100 |
| **PR32** | I agreed with the purpose of the project | 4 | 100 |
| **PR34** | Before I agreed to be a patient partner, the researcher clearly explained all aspects of the project | 4 | 91 |
| **PR35** | I was provided with regular updates on the project | 4 | 91 |
| **PR36** | The research team communicated in language that was understandable to me | 4 | 91 |
| **PR39** | I understood the research goals | 4 | 100 |
| **Convenience (n=5)** | | **4** |  |
| **CVN1** | I was involved in choosing my research related tasks | 4 | 82 |
| **CVN3** | My preferences for timing, duration, format, and access of scheduled meetings were considered | 4 | 91 |
| **CVN4** | I met with the research team at times that were convenient to me | 4 | 91 |
| **CVN7** | I was given enough time to contribute to the project | 4 | 100 |
| **CVN9** | I had opportunities to express my views | 4 | 100 |
| **Contributions (n=4)** | |  |  |
| **CTB5** | I contributed by providing my perspective | 4 | 100 |
| **CTB11** | My contributions were a good use of my time | 4 | 100 |
| **CTB15** | The other team members and I shared knowledge and learned from each other | 4 | 82 |
| **CTB16** | My workload in the project was manageable | 4 | 100 |
| **Research Environment (n=2)** | |  |  |
| **RE2** | Throughout the project, I felt accepted as a member of the research team | 4 | 91 |
| **RE3** | I was treated as an equal by the other members of the research team | 4 | 82 |
| **Team Interaction (n=3)** | |  |  |
| **TI3** | My interactions within the research team were positive | 4 | 100 |
| **TI6** | I felt there was mutual respect within the research team | 4 | 91 |
| **TI9** | There was trust among the research team members | 4 | 100 |
| **Support (n=3)** | |  |  |
| **SU5** | I received the training I needed for my role | 4 | 100 |
| **SU6** | I had access to the resources I needed | 4 | 100 |
| **SU7** | I received reimbursement for out-of-pocket expenses (such as for childcare, parking, and travel) | 4 | 100 |
| **Feel Valued (n=3)** | |  |  |
| **FV2** | My contributions were appreciated | 4 | 82 |
| **FV8** | My contributions were valued by the research team | 4 | 100 |
| **FV9** | There was a general openness to receiving my views | 4 | 91 |
| **Benefits (n=2)** | |  |  |
| **BE3** | I enjoyed being involved | 4 | 82 |
| **BE8** | I see how my contributions could benefit other people | 4 | 100 |

**Items that partially met the selection criteria**

| **Code** | **Item** | **Median** | **% score 3 and 4** |
| --- | --- | --- | --- |
| **Procedural Requirements (n=14)** | |  |  |
| **PR1** | The project was suited to my interests | 3 | 91 |
| **PR2** | The project matched my experiences | 3 | 100 |
| **PR4** | Unfamiliar terms or acronyms were explained to me by the research team | 3 | 91 |
| **PR5** | The project included views of patient partners from different personal backgrounds if applicable [such as age, gender, education level, economic class, and disease diagnosis] | 3 | 82 |
| **PR11** | I shared information from the patient community with the research team | 3 | 91 |
| **PR20** | My ability to contribute was not affected by team changes | 3 | 80 |
| **PR16** | My experiences were a ‘good match’ for what was needed for the research | 3 | 100 |
| **PR15** | I received payment for my effort and contributions | 3 | 72.7 |
| **PR24** | I understood the role of the other research team members | 3 | 82 |
| **PR29** | I had regular communication with the rest of the research team | 3 | 82 |
| **PR31** | I was involved throughout the whole project | 3 | 72.7 |
| **PR41** | We had two-way communication during our conversations | 3 | 100 |
| **PR42** | I was given constructive feedback on my contributions | 3 | 82 |
| **PR43** | I had opportunities to provide feedback about my experiences on the project | 3 | 82 |
| **Convenience (n=1)** | |  |  |
| **CVN8** | Team meetings were conveniently scheduled | 3 | 100 |
| **Contributions (n=6)** | |  |  |
| **CTB4** | I provided lay language to communicate our research | 3 | 72.7 |
| **CTB8** | I contributed by using my critical thinking skills | 3 | 72.7 |
| **CTB9** | I felt confident in my ability to contribute | 3 | 91 |
| **CTB10** | I contributed by using my professional or personal skills | 3 | 82 |
| **CTB13** | My role was important for conducting the research | 3 | 91 |
| **CTB14** | My knowledge was valuable to the project | 3 | 90 |
| **Research Environment(n=3)** | |  |  |
| **RE1** | Throughout the project, I felt comfortable as a member of the research team | 3 | 91 |
| **RE4** | Other team members were welcoming | 3 | 82 |
| **RE5** | I had the option of joining meetings remotely | 3 | 100 |
| **Team Interactions(n=4)** | |  |  |
| **TI4** | I was satisfied with the amount of interaction I had with the research team | 3 | 100 |
| **TI5** | I felt comfortable talking with members of the research team | 3 | 82 |
| **TI10** | I found the research team members to be reliable | 3 | 91 |
| **TI12** | I had one-on-one communication with the researcher(s) | 3 | 72.7 |
| **Support(n=2)** | |  |  |
| **SU1** | I received an appropriate amount of support for my contributions | 3 | 91 |
| **SU4** | My concerns were addressed | 3 | 100 |
| **Feel Valued(n=7)** | |  |  |
| **FV1** | My contributions were acknowledged | 3 | 91 |
| **FV3** | I felt I was an important team member | 3 | 91 |
| **FV5** | I was treated as a member of the research team | 3 | 82 |
| **FV6** | I was respected within the research team | 3 | 100 |
| **FV7** | Members of the research team respected my views | 3 | 91 |
| **FV10** | My contributions were used | 3 | 91 |
| **FV12** | I was paid for my contributions | 4 | 64 |
| **Benefits(n=9)** | |  |  |
| **BE2** | I gained or improved my skills | 3 | 72.7 |
| **BE4** | I felt more empowered | 3 | 72.7 |
| **BE5** | I gained more confidence about my research knowledge and skills | 3 | 91 |
| **BE6** | I gained new opportunities to do activities I am interested in | 3 | 82 |
| **BE9** | My involvement in decision making had an impact on the project | 3 | 91 |
| **BE10** | I benefited from talking with other patients/families/caregivers about the project | 3 | 72.7 |
| **BE13** | I learned from my engagement in the project | 3 | 82 |
| **BE15** | I had the opportunity to present at research conferences | 3 | 82 |
| **BE16** | My involvement had positive impacts on my life. | 3 | 82 |

**Items that did not meet the selection criteria**

| **Code** | **Item** | **Median** | **% score 3 and 4** |
| --- | --- | --- | --- |
| **Benefits (n=1)** | |  |  |
| **BE11** | I benefited from interacting with the public about the research | 3 | 63.6 |

**Items rated on whether or not they should be kept that met the selection criteria**

| **Code** | **Item** | **% Yes** |
| --- | --- | --- |
| **Procedural Requirements (n=1)** | |  |
| PR27 | I understood the responsibilities of the research organization and research team members | 55 |

*PR27 met the selection criteria of >50% of participant rating that it should be kept in the questionnaire.

**Items rated on whether or not they should be kept that did not meet the selection criteria**

| **Code** | **Item** | **% Yes** |
| --- | --- | --- |
| **Procedural Requirements (n=11)** | |  |
| **PR8** | The researcher(s) ensured contributions were suitable for the project before using them | 0 |
| **PR9** | I became gradually more involved in the project | 9 |
| **PR10** | I was involved from the start of the project | 36 |
| **PR17** | The researchers and I had different roles in the project | 9 |
| **PR19** | I shared tasks with other research team members | 18 |
| **PR25** | I valued the role of the other research team members | 30 |
| **PR28** | I communicated on a regular basis with the researcher(s) | 27 |
| **PR30** | My private information was kept confidential | 36 |
| **PR33** | I had adequate opportunities to be involved | 27 |
| **PR37** | There understood the rules regarding sharing of project results | 18 |
| **PR38** | All members of the team agreed the study was important | 9 |
| **Convenience (n=3)** | |  |
| **CVN2** | I had the flexibility to do less or stop doing tasks | 36 |
| **CVN5** | The format of the meetings met my needs | 18 |
| **CVN6** | Things that needlessly restricted my ability to contribute were changed | 27 |
| **Contributions (n=6)** | |  |
| **CTB1** | I contributed to the governance of the project | 27 |
| **CTB2** | I contributed to the project from its beginning to end | 18 |
| **CTB3** | My contributions led to the successful completion of the project | 0 |
| **CTB6** | I shared my experience of having my disease/condition | 27 |
| **CTB7** | I was willing to contribute regardless of any uncertainties | 9 |
| **CTB12** | I was willing to contribute without compensation | 9 |
| **Team Interaction (n=5)** | |  |
| **TI1** | I built friendships with the research team members | 0 |
| **TI2** | I was encouraged to contribute | 36 |
| **TI7** | The researcher(s) mentored me | 9 |
| **TI8** | I socialized with the other research team members | 0 |
| **TI11** | The team as a whole met face-to-face on occasion | 18 |
| **Support (n=2)** | |  |
| **SU2** | A research team member helped me with my contributions to the project | 0 |
| **SU3** | Other patient research partners helped me to provide my contributions | 9 |
| **Feel Valued (n=2)** | |  |
| **FV4** | My viewpoints were addressed | 36 |
| **FV13** | I received non-financial gifts for my contribution | 0 |
| **Benefits (n=4)** | |  |
| **BE1** | I gained free access to academic peer-reviewed journal articles | 36 |
| **BE7** | I gained a renewed purpose in life | 0 |
| **BE12** | I have learned more about my disease and its treatment | 18 |
| **BE14** | I had the opportunity to attend research conferences | 45 |
