# Supplementary material for: Development and pre-testing of the Patient Engagement In Research Scale (PEIRS) to assess the quality of engagement from a patient perspective
Source: PLoS One. 2018 Nov 1;13(11):e0206588. doi: 10.1371/journal.pone.0206588 (PMC6211727; doi:10.1371/journal.pone.0206588)
Supplement: S2 Appendix — (DOCX) [file pone.0206588.s003.docx]

**S2 Appendix** Per-item summary of the ratings for level of importance in round three of the Delphi survey

The items are categorized based on the two quantitative selection criteria. A third, supplementary, selection criteria was participants’ comments. The final decision to select an item was based on our research team discussion and consensus.

**Items that met the selection criteria**

| **Code** | **Item** | **Median** | **% score 3 and 4** |
| --- | --- | --- | --- |
| **Procedural Requirements (12 items)** | |  |  |
| **PR3** | The project was worth the time I spent on it | 4 | 100 |
| **PR7** | I had opportunities to contribute within the research team | 4 | 91.7 |
| **PR14** | The research team members were properly introduced to each other | 3.5 | 91.7 |
| **PR18** | I participated in making decisions about the project | 3.5 | 100 |
| **PR21** | There was an appropriate number of patient partners | 4 | 90.9 |
| **PR22** | I had a clear understanding of my role | 4 | 91.7 |
| **PR23** | I understood the expectations of my contributions to the project | 4 | 100 |
| **PR26** | I was interested in the issue(s) being researched in the project | 4 | 83.3 |
| **PR32** | I agreed with the purpose of the project | 3.5 | 91.7 |
| **PR35** | I received sufficient updates on the project | 4 | 91.7 |
| **PR36** | We had clear communication within the research team | 4 | 100 |
| **PR39** | I understood the research goals | 4 | 100 |
| **Convenience (2 items)** | |  |  |
| **CVN7** | I had sufficient time to complete my tasks for the project | 4 | 91.7 |
| **CVN9** | I had opportunities to express my views | 4 | 91.7 |
| **Contributions (4 items)** | |  |  |
| **CTB5** | I contributed by providing my perspective as a research partner | 4 | 100 |
| **CTB11** | I feel my contributions were a good use of my time | 4 | 83.3 |
| **CTB15** | The other team members and I shared our knowledge | 3.5 | 83.3 |
| **CTB16** | I feel my workload in the project was manageable | 4 | 83.3 |
| **Research Environment (2 items)** | |  |  |
| **RE2** | Throughout the project, I felt accepted as a member of the research team | 4 | 100 |
| **RE3** | I feel that I was an equal partner in the research team | 4 | 83.3 |
| **Team Interaction (3 items)** | |  |  |
| **TI3** | My interactions within the research team were positive | 4 | 83.3 |
| **TI6** | I feel that there was mutual respect among the research team members | 4 | 100 |
| **TI9** | I feel that there was trust among the research team members | 4 | 100 |
| **Support (5 items)** | |  |  |
| **SU1** | I received sufficient support to contribute to the project | 4 | 81.8 |
| **SU4** | My concerns were addressed | 3.5 | 83.3 |
| **SU5** | I received the training I needed for my role | 4 | 91.7 |
| **SU6** | I had access to both financial and non-financial resources for my tasks in the project | 4 | 100 |
| **SU7** | I was offered sufficient reimbursement for my out-of-pocket expenses (such as for childcare, parking, and travel) due to the project activities | 4 | 100 |
| **Feel Valued (4 items)** | |  |  |
| **FV2** | I feel that the research team appreciated my contributions | 4 | 91.7 |
| **FV8** | I feel that the research team valued my contributions | 4 | 91.7 |
| **FV9** | I feel that the research team had a general openness to receiving my views | 4 | 100 |
| **FV12** | I was offered sufficient compensation for my contributions | 3.5 | 75 |
| **Benefits (2 items)** | |  |  |
| **BE3** | I enjoyed being a part of the project | 3.5 | 83.3 |
| **BE8** | I see how my contributions could benefit other people | 3.5 | 91.7 |

**Items that partially met the selection criteria**

| **Code** | **Item** | **Median** | **% score 3 and 4** |
| --- | --- | --- | --- |
| **Procedural Requirements ( 8 items)** | |  |  |
| PR6 | I understood the ethical requirements for the project | 3 | 87.3 |
| PR12 | My tasks in the project fit with my abilities | 3 | 91.7 |
| PR13 | My tasks in the project fit with my availability | 3 | 100 |
| PR16 | My experiences were useful for the project | 3 | 75 |
| PR27 | I understood the responsibilities of the research team members | 3 | 91.7 |
| PR29 | I had sufficient communication with the research team about the project | 3.5 | 66.7 |
| PR34 | I received sufficient explanation on all aspects of the project | 3 | 83.3 |
| PR43 | Throughout the project, I had opportunities to provide feedback about being a part of the research team | 3.5 | 66.7 |
| **Convenience (2 items)** | |  |  |
| CVN1 | I helped to choose my research related tasks | 3 | 75 |
| CVN3 | My preferences for meetings (such as time, duration, location, and format) were considered when planning meetings | 3 | 100 |
| **Contributions (1 item)** | |  |  |
| CTB14 | I feel that my knowledge was valuable to the project | 3 | 83.3 |
| **Research Environment (1 item)** | |  |  |
| RE5 | I had the option of joining meetings remotely | 4 | 66.7 |
| **Feel Valued (1 item)** | |  |  |
| FV1 | My contributions were acknowledged | 3 | 83.3 |
| **Benefit (3 items)** | |  |  |
| BE2 | I gained or improved my knowledge through engaging in the project | 3 | 83.3 |
| BE9 | I feel that my involvement in decision making had an impact on the project | 3 | 75 |
| BE16 | My involvement had positive impacts on my life. | 4 | 63.6 |

**Items that did not meet the selection criteria**

| **Variable** | **Item** | **Median** | **% score 3 and 4** |
| --- | --- | --- | --- |
| **Procedural Requirements (2 items)** | |  |  |
| PR1 | The project piqued my interest | 3 | 63.6 |
| PR42 | I received constructive feedback on my contributions | 3 | 66.7 |
| **Contributions (1 item)** | |  |  |
| CTB13 | I feel that I played an important role in the project | 3 | 58.3 |
| **Research Environment (1 item)** | |  |  |
| RE1 | Throughout the project, I felt comfortable as a member of the research team | 3 | 66.7 |
| **Team Interaction (1 item)** | |  |  |
| TI4 | I feel satisfied with the amount of interaction I had with the research team | 3 | 66.7 |
| **Benefits (2 items)** | |  |  |
| BE13 | I learned from my engagement in the project | 3 | 66.7 |
| BE15 | I had the opportunity to attend research conferences | 3 | 58.7 |
